# Pesticides in soil, groundwater and food in Latin America as part of one health

**DOI:** 10.1007/s11356-024-32036-3

**Published:** 2024-02-08

**Authors:** Isabel Hilber, Fernando Bahena-Juárez, Aurea C. Chiaia-Hernández, Sebastián Elgueta, Arturo Escobar-Medina, Karen Friedrich, Miguel Ángel González-Curbelo, Yael Grob, Marisleydis Martín-Fleitas, Karina S. B. Miglioranza, Brizeidi Peña-Suárez, Nilda Pérez-Consuegra, Fernando Ramírez-Muñoz, Dayana Sosa-Pacheco, Thomas D. Bucheli

**Affiliations:** 1 Environmental Analytics, Agroscope, Zurich, Switzerland; 2 Campo Experimental Uruapan, CIRPAC, INIFAP, Uruapan, Mexico; 3 Institute of Geography and Oeschger Center for Climate Change Research, University of Bern, Bern, Switzerland; 4 Núcleo en Ciencias Ambientales y Alimentarias (NCAA), Universidad de Las Américas, Providencia, seat Santiago, Chile; 5 Centro Nacional de Sanidad Agropecuaria, San José de Las Lajas, Cuba; 6 Centro de Estudios y Salud del Trabajador y Ecología Humana, Escuela Nacional de Salud Pública Sergio Arouca, Fundación Oswaldo Cruz, Rio de Janeiro, Brazil; 7 Departamento de Ciencias Básicas, Facultad de Ingeniería, Universidad EAN, Bogota, Colombia; 8 Empresa de Aprovechamiento Hidráulico de Mayabeque, Havana, Cuba; 9 Instituto de Investigaciones Marinas y Costeras, Universidad Nacional de Mar del Plata, Mar del Plata, Argentina; 10 Universidad Agraria de La Habana “Fructuoso Rodríguez Pérez”, San José de Las Lajas, Cuba; 11 Instituto Regional de Estudios en Sustancias Tóxicas, Universidad Nacional, Heredia, Costa Rica

**Keywords:** Pesticides, Environmental and human exposure and risks, Actionable research, Science-policy interface

## Abstract

We here report of a conference about “Pesticides in Soil, Groundwater and Food in Latin America as part of One Health” that took place at the “IV Seminario Internacional de Sanidad Agropecuaria (SISA)” in Varadero, Cuba, 8–12 May 2023. Researchers of Latin America (Argentina, Brazil, Chile, Costa Rica, Colombia, Cuba, Mexico) and Switzerland (workshop initiator) held presentations about occurrence and effects of pesticides on the environment, human health, the replacement of highly hazardous pesticides (HHP) by agroecological alternatives and the agri-food value chain. In a subsequent round table discussion, the presenters identified deficits, needs, interests and opportunities. According to them, the lack of awareness of pesticide use affects the health and safety of workers applying the chemicals. Despite Latin America representing the main agricultural area in the world with a very intense pesticide use, monitoring data of pesticides in soil, surface and groundwaters, food, as well as in humans are missing. Risks of pesticides to humans should be assessed so that authorities can withdraw or limit within “short time” the access to corresponding formulations on the market. Also, communication is not state of the art and should be improved as, e.g. the teaching of workers and farmers, how to correctly use and apply pesticides or the briefing of decision makers. Pollinators suffer from multiple stressors not the least due to pesticides, and alternatives are badly needed. On the technical side, the different analytical methods to determine residues of active ingredients and transformation products in matrices of concern should be harmonized among laboratories.

Seven future actions and goals were identified to overcome the above deficits. Next steps after the publishing of this conference report are to harmonize and complete the information status of the presenters by exchanging the results/data already present. Therefore, a platform of interaction to address issues described above and to enhance collaboration shall be created. Samples of different matrices shall be exchanged to harmonize the chemical analysis and establish interlaboratory comparisons. Such activities might be facilitated by joining international associations or organizations, where researchers can offer their expertise, or by forming a new pesticide network for Central and South America that could present tailored projects to national and international organizations and funding agencies.

## Evolution of and contributions to the pesticide conference

**Fig. 1:**
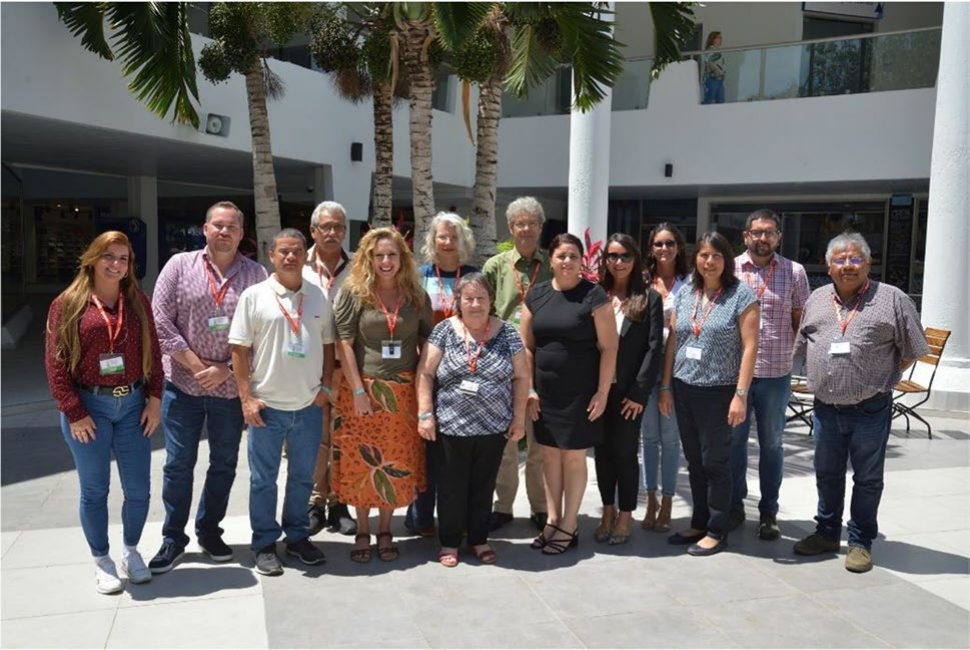
Scientists who contributed to the workshop “Pesticides in soil, groundwater and food in Latin America as part of one health” at the IV International Seminar on Animal and Plant Health (SISA) in Varadero, Cuba, 8–12 May 2023. From the left: Yanna Llerena-Padrón (non-presenter), Miguel Ángel González-Curbelo, Fernando Ramírez-Muñoz, Arturo Escobar-Medina, Karen Friedrich, Isabel Hilber, Nilda Pérez-Consuegra, Thomas D. Bucheli, Dayana Sosa-Pacheco, Karina S. B. Miglioranza, Brizeidi Peña-Suárez, Aurea C. Chiaia-Hernández, Sebastián Elgueta and Fernando Bahena-Juárez. Missing: Yael Grob and Marisleydis Martín-Fleitas

We here report on a two-day workshop with the title “Pesticides in Soil, Groundwater and Food in Latin America as Part of One Health” that took place as part of the IV International Seminar on Animal and Plant Health (SISA), in Varadero, Cuba, 8–12 May 2023. The workshop had the objectives, firstly, to present the outcomes of the five-year project PERECUSO (pesticide residues in Cuban soils, IZ08Z0_177481/1) (Agroscope 2019a, b, Swiss National Science Foundation 2023; Staubesand 2021; Telecentro Provincial Telemayabeque 2019), which ended in September 2023 and focused on pesticide residues in soil and potato tubers from the Mayabeque Province in Cuba. PERECUSO was financed by the Swiss National Science Foundation (SNSF). Secondly, the workshop aimed at bringing together a number of Latin American scientists and experts in the field (Fig. 1) who supported PERECUSO over the years and contributed substantially to the workshop with their expertise.

Different research and ongoing studies related to pesticides in Latin America were presented including the effects of pesticides on the environment and human health, the replacement of highly hazardous pesticides (HHP) by agroecological alternatives and the agri-food value chain. Some of these data included pesticide residues and degradation products gathered in various matrices, including (i) organically or conventionally managed agricultural soils and corresponding mass balances for some active ingredients, (ii) sediment, (iii) food and (iv) ground- and surface waters. The titles, highlights and presenters of these contributions are listed in Table 1.

**Table 1:** Scientific contributions to the two-day workshop “Pesticides in soil, groundwater and food in Latin America as part of one health” at the IV International Seminar on Animal and Plant Health (SISA) in Varadero, Cuba, 8–12 May 2023, and main findings therefrom

Researcher	Title of presentation and main findings
Bahena-Juárez Fernando, Mexico	**Replacement of highly hazardous pesticides (HHPs) with agroecological alternatives in maize production in Mexican agriculture** Agroecological alternatives were presented to substitute HHP applied to corn crops. Agroecological pest management proposes simultaneous actions of conservation agriculture and crop harvest residue incorporation, direct sampling of pests in crops, use of biopesticides and natural insecticides from plant extracts, ethological control with traps and sexual pheromones, biological control applied with the release of beneficial insects and the restoration of functional biodiversity to reverse monoculture (Barrientos 2021).
Bucheli Thomas, Switzerland	**Pesticides in agricultural soils: major findings from various monitoring campaigns in Switzerland** Studies this far revealed that (1) multiple pesticides are commonly present in soils, with individual concentrations in agricultural soils often reaching up to a few tens of micrograms per kilogram; (2) pesticide occurrence and concentrations in agricultural soils primarily depend, among other influencing factors, on land use and cultivated crops, as they require different types of active ingredients, amounts and frequencies of application; (3) pesticides can prevail much longer than predicted by their half-lives and were found in soils even decades after conversion from conventional to organic farming (Riedo et al. 2021). Corresponding residual fractions can be in the order of a few percent of the originally applied amounts (Riedo et al. 2023). Traces of pesticides are also detected in soils to which they were never applied, indicating contamination, e.g. via spray drift or atmospheric deposition (Riedo et al. 2022).
Hilber Isabel, Switzerland	**Losses of pesticides via drainage over one growing season on a potato field** Eighteen active ingredients were applied on a potato field in Zurich, Switzerland, during April to August 2020. Twenty-one active ingredients incl. seven metabolites were found in the drainage water of the very same field in a highly dynamic concentration range with a maximum of up to a few tens of micrograms per litre. In other studies, similar concentrations were found as for instance in surface and/or groundwaters of rivers of Spain and Hungary (Borrull et al. 2019; Tölgyesi et al. 2022; Tóth et al. 2022). Exported fractions of total applied amounts were < 1%, and the groundwater ubiquity score (GUS), composing of organic carbon to water partition coefficient (*K*_OC_) and the pesticide’s half-life (*DT*_50_), predicted this export well. The comparison of the exported fractions and max. concentrations with data from the EXPOSIT model, used in Switzerland’s pesticide authorization to forecast surface water concentrations, revealed that predicted occurrences of pesticides in surface waters are robust and conservative. Atrazine, last applied in 2009, and its degradation product atrazine-2-hydroxy leached over the whole experiment duration (corresponding paper submitted to Environmental Science & Technology).
Chiaia-Hernández Aurea, Switzerland	**Tracing pesticide sources in urban receiving water based on agricultural activities fingerprint** Even though pesticides have been studied for decades in different matrices (e.g. plants, food and water), not much is known about their fate and occurrence in sediments. The results presented at SISA 2023 show how multiproxy workflows based on paleolimnology tools and liquid chromatography coupled to tandem mass spectroscopy can open opportunities to study retrospectively the contamination of catchments with different pesticides starting in the 1960s based on the analysis of sediment cores from lake systems (Chiaia-Hernández et al. 2020b). Additionally, the results presented demonstrate that, once deposited in sediment, pesticides are often quite stable, especially under anaerobic conditions and concentrations and fluxes over time are related only to pesticide application (inferred from sales) or regulatory measures (bans) and not to terrestrial processes (e.g. soil erosion or lake biogeochemistry). The data discussed also correlates with current soil and groundwater monitoring which highlights the use of sediments as archives of environmental contaminants (Chiaia-Hernández et al. 2020a, 2020b).
González-Curbelo Miguel Ángel, Colombia	**Pesticide residues in Colombia: distribution, levels and potential risk to ecosystems and human health** Colombia has played a leading role in the production and marketing of pesticides in recent decades. In addition, it ranks 18^th^ among the top pesticide users worldwide (FAOSTAT 2020). Regarding active ingredients, glyphosate (14%), chlorpyrifos (7.5%) and mancozeb (6.9%) have been the top three most sold (Instituto Colombiano Agropecuario 2021). Despite the above findings, there is no consolidated knowledge of the overall related risks. Thus, the National Plan for Surveillance and Control of Pesticide Residues has been developed to determine and quantify the presence of pesticide residues in foods of plant origin in primary production. The first results were reported in 437 banana samples from two departments of Colombia (Magdalena and Antioquia). The results indicated that 50% of the samples presented pesticide residues (azoxystrobin, boscalid, chlorpyrifos, fenpropimorph, myclobutanil, pyrimethanil, pyriproxyfen and thiabendazole of 94 pesticides analysed), but only for chlorpyrifos, the maximum residue limit was exceeded (Instituto Colombiano Agropecuario 2022a). Subsequent studies collected 1107 samples of rice, corn, tomato, onion, potato, leafy vegetables, beans, peas, strawberry, blackberry, granadilla and passion fruit to determine 320 pesticide residues, and results will be published during the first semester of 2023 (ICA 2022b). Meanwhile, residues of chlorpyrifos, chlorpyrifos-methyl, fenitrothion and diphenylamine (Varela-Martínez et al. 2019), as well as propoxur and propyzamide (Varela-Martínez et al. 2020) have been recently reported in minor tropical fruits, but all of them were at concentrations below the maximum residue limit.
Ramírez-Muñoz Fernando, Costa Rica	**Chlorothalonil fungicide as a contaminant of drinking water sources in horticultural production areas of Costa Rica** Chlorothalonil is a fungicide applied aerially in Costa Rica in extensive crops, such as export bananas, and also in countless vegetables for national consumption such as potatoes, onions, carrots, celery and others, where it is one of the main pesticides used. High quantities (in kg_active ingredients_/ha/cycle) are used in banana plantations (7), onion (11), potato (8), tomato (5), pepper and carrot (4) and many others. Frequent applications in cultivation areas have caused soil microorganisms to evolve, degrade and form more soluble, toxic and persistent metabolites, which can leach into groundwater, contaminating drinking water sources for many populations. Chlorothalonil can be transported from the application sites in low areas by evaporation and atmospheric transfer until it reaches higher and cooler areas where it condenses and impacts non-agricultural areas such as cloud forests in National Parks, wildlife and being one of the candidates for the disappearance of frog species (Shunthirasingham et al. 2011). Similarly, chlorothalonil has been found for several decades contaminating the air, soil, surface water, crops and food, thereby negatively impacting the overall ecosystem health. We require that this pesticide be banned in Costa Rica, as it currently is in many countries globally.
Pérez-Consuegra Nilda, Cuba	**Update of pesticides in Cuba with emphasis on productive agroecosystems and implications for health (PERECUSO project)** The use of HHP in Cuba poses risks to human health, animals, plants and the overall ecosystem. Three hundred and forty active ingredients were identified in 877 authorized pesticide formulations, of which 110 (32.6%) are HHP according to the World Health Organization (WHO) and Pesticide Action Network (RAP-AL) classification. Some active ingredients exhibit acute and chronic toxicity and are carcinogenic (e.g. abamectin, benomyl, cyfluthrin, chlorothalonil, oxamyl, mancozeb). Additionally, active ingredients with high environmental toxicity including to pollinators are authorized. While it is believed that the small quantities of pesticides used in Cuba do not pose a danger to bees, it is important to pay attention to this issue. Cuba has been promoting a sustainable agricultural model based on agroecology. Policies and laws related to agroecology, bioproduct production and food sovereignty have been approved. These measures aim to reduce the relevance of HHP and promote safer and more sustainable agricultural practices.
Escobar-Medina Arturo, Cuba	**Pesticide residues in potato (*Solanum tuberosum* L.) of Mayabeque, Cuba (PERECUSO Project)** Nineteen potato producer sites (18 conventionally, one organically managed for 10 years) were sampled in Mayabeque, Cuba between 2018 and 2022. Thirty-one currently used pesticides (39% fungicides, 24% herbicides, 18% insecticides/acaricides) specified in the official technical instructions for potato production in Cuba, and seven transformation products were analysed in soil (*n* = 152) and potato samples (*n* = 50). In 93% of the soil samples, residues of ≥1 active ingredient and/or transformation product were detected. Median values of all compounds and samples oscillated between a few of up to a few hundred micrograms per kilogram in soil. In potato samples, 11 active ingredients and transformation products were quantified in 50% of the samples. Mean values of the sum of compounds ranged from a few hundredths (0.01) up to a few tens (10) of micrograms per kilogram of fresh weight potato. (Corresponding paper in preparation.)
Peña-Suárez Brizeidi, Cuba	**Pesticide residues in soils in relation to pesticide application in Mayabeque, Cuba (PERECUSO Project)** This is the first study that connects application records of farmers with residues and dissipation (*DT*_50_) of currently used pesticides in Cuban agricultural soils. A subset of the above-mentioned soil samples (by Arturo Escobar-Medina), with complete application data, were taken from six conventionally managed sites (Mayabeque, Cuba) between 2018 and 2021 at three times per year (before planting potato, at high pesticide application time and at harvest (6 sites × 3 years × 3 sampling times = 54). Sixty-three percent of 31 active ingredients and seven transformation products (see also above) were found in soil samples. Although fungicides were predominantly applied, two herbicides were mostly detected in the soil samples. With the concentrations found in soil and the application information, field *DT*_50_ were calculated and compared with the pesticides properties database (University of Hertfordshire 2023). For one active ingredient, the half-lives calculated under Cuban soil conditions (*DT*_50obs_) were lower than in temperate climate, while for another, they were quite similar. (Corresponding paper in preparation.)
Sosa-Pacheco Dayana, Cuba	**Pesticide residues in groundwaters of Mayabeque, Cuba in relation to their respective agricultural soils (CUBAGUA)** The main objectives of this study were (i) to evaluate the presence of pesticides and transformation products in groundwaters of the Mayabeque province, Cuba and (ii) to relate the findings of the PERECUSO project (above and below), thus, pesticides applied and residues found in soils of the same province, with the ones of CUBAGUA. For that, water wells of the Cuban water quality network (RedCal, see contribution below of Marisleydis Martín-Fleitas, Cuba) in Quivicán (*n* = 18) and Batabanó (*n* = 8) were sampled according to the RedCal protocol during the dry and wet season in 2022. Individual pesticide concentrations in groundwater samples were a few to < 100 ng/L, thereby being in the same range as other authors reported (Grondona et al. 2023; Tölgyesi et al. 2022). Only 4% (*n* = 2) of all samples (*n* = 52) had pesticide concentrations that surpassed the maximum residue level established by the European Directive. All active ingredients detected in soils were also detected in groundwater. Active ingredients not applied and not detected in soil but found in groundwaters reflected the memory of soil. These results shed first light on the quality of Cuban groundwaters and thereby serve as a basis for a risk assessment of human health in Cuba. (Corresponding papers in preparation.)
Elgueta Sebastián, Chile	**Pesticide residues in plant products and consumer risk in the agri-food value chain in Chile** The Information Network and Food Alerts Program in Chile (RIAL) has published serious transgressions of maximum residue limits of pesticide residues mainly in fresh vegetables in the last five years (Aylwin 2018, 2019, 2020). In addition, the National System of Monitoring of Acute Pesticide Poisonings (Stagno 2018) stated that from the total cases reported between 2012 and 2018, 50% were acute pesticide poisonings caused by cypermethrin, chlorpyrifos, lambda-cyhalothrin, methamidophos, diazinon, alpha-cypermethrin, glyphosate, azinphos-methyl, deltamethrin, methomyl and paraquat. In the period of 2012 to 2018, 3914 cases of acute poisonings were reported and 35% of them were deaths caused by pesticides extremely toxic and toxic (Ia and Ib) according to the WHO (2020). Therefore, the post-registration process of pesticides must be improved including national surveillance and environmental and human risk assessment in matrices such as soil, water and food. The decision-making of post-registration should consider scientific information and the precautionary principle approach for rapid actions to reduce the risks to human health and remove HHP from the Chilean market.
Miglioranza Karina, Argentina	**Pesticides in agriculture: research in Latin America** According to the WHO, air pollution is an environmental problem that has increased in recent years, mainly in large urban conglomerates, and is the main environmental health risk in Latin America. The emission of contaminants into the atmosphere such as persistent organic pollutants (POPs) and currently used pesticides is a problem of great interest due to their toxicity, persistence and capacity to be transported and accumulated in organisms. POPs include organochlorine pesticides (OCPs), polychlorinated biphenyls (PCBs) and polybrominated diphenyl ethers (PBDEs), among others, which are compounds with a long half-life, in some cases reaching more than 30 years in soils. They are ubiquitous, hydrophobic, with chronic toxicity, are banned worldwide, and regulated by the Stockholm Convention (Stockholm Convention on Persistent Organic Pollutants 2011). Due to their volatility, they can be transported with air masses reaching distant regions through dry or wet depositions (precipitation). The large agricultural expansion in Latin America leads to an intense use of agrochemicals, with Argentina being one of the main soybean producers and the country with the highest consumption of glyphosate per unit of cultivated area. Residues of these pesticides are found in different matrices of aquatic and terrestrial ecosystems, rainwater and air, reaching places beyond the application site (González Noschese et al. 2022; Miglioranza et al. 2021; Quadri-Adrogué et al. 2021; Silva-Barni et al. 2019; Villalba et al. 2020). There is a need to change the paradigm of indiscriminate and poorly controlled use of chemical pesticides in order to achieve sustained sustainability over time.
Friedrich Karen, Brazil	**Use and regulation of pesticides in Brazil and their impact on human health** The risk assessment of pesticides presents several limitations such as studies performed with one route and one active substance at once, ignoring the potential of synergism of mixtures of pesticides and other chemical substances used in agriculture. Toxic effects may be expressed in various and complex physiological functions that might not be captured in the risk assessment or the study. Dose–response curves may not be linear, thereby hampering safety limits determination. The existence of critical periods of organism development may impose hazards not identified in toxicological studies used for risk assessment. Besides that, safety conditions are often not met when pesticides are applied, such as not putting personal protective equipment (PPE). Pesticides in water and food should be monitored as well as exposed populations kept under surveillance. Pesticides in Brazil should be monitored regularly in water, food and other matrices according to Brazilian regulation. However, few data are available about pesticide residues and the major limitation are scarcely capable and available laboratories to perform these analyses. Conditions of safety determined by risk assessors demand adequate data to assess the profile of exposure in Brazilian population and if regulatory limits are being followed.
Martín-Fleitas Marisleydis, Cuba	**RedCal, the water quality net: importance of compliance** Water can transport, amongst others, contaminants that affect the human health. These contaminants can originate from industries or from agriculture such as pesticides. As nowadays still people die by polluted drinking water, it is important to monitor its quality. The Cuban water quality network RedCal in the Mayabeque Province composes of 176 stations of calcic bicarbonate character (Farfán et al. 2014) and takes about 488 water samples annually to check their physical, chemical and biological parameters. Those waterholes supplying the population are sampled monthly and sent to the RedCal laboratory. The management of hydraulic exploitation in Mayabeque analyses and compares the lab results with the Cuban norms to ascertain the water quality is within the parameters and, if not, to search for (a) cause(s). Measures to eliminate irregularities are planned and actions taken. Furthermore, the results are registered in a database to compare the state of the waters over time and space, to recognize trends and possible alterations of parameters. RedCal is of vital importance when it comes to good water quality and, with that, to an adequate human health. It therefore complies with the United Nation’s Sustainable Development Goals.
Peña Suárez Brizeidi, Cuba	**Validation of a multi-residual method for pesticide analysis in soil and potato by gas chromatography tandem mass spectrometry (PERECUSO Project)** An analytical method to detect and quantify pesticide residues in tropical agricultural soils was established and validated according to the latest SANTE guideline (2020). Thirty-one currently used pesticides (commonly used in Cuban agriculture) and seven transformation products, were extracted by the QuEChERS (Quick, Easy, Cheap, Effective, Rugged and Safe) method and analysed by gas chromatography coupled to tandem mass spectrometry. Matrix effects were studied for five different Cuban soils and 10 isotopically labelled internal standards used in matrix matched calibrations to compensate, amongst others, this effect. All analytical parameters (matrix effects, repeatability and reproducibility (as measures of precision), recoveries, limit of quantification, linearity of matrix matched calibration curves and specificity and selectivity) proved to be satisfactory according to the SANTE guideline (2020) for a Cuban forestry soil free of pesticides. The method was successfully applied to 30 agricultural soil samples. Twenty-four currently used pesticides and five transformation products were detected in a range between a few of up to a few hundred micrograms per kilogram in soil. These data serve as a basis for future soil monitoring programs of currently applied pesticides in Cuba. (Corresponding paper submitted to Environmental Science and Pollution Research.)
Grob Yael, Switzerland	**Monitoring of pesticide residues in a potato field in Switzerland (PERECUSO Project)** Eighteen active ingredients were applied on a potato field in Zurich, Switzerland, during April to August 2020. Out of 20 active ingredients applied, 21 were analysed and 17 found in the topsoil (0–5 cm) after their first application. Additionally, eight metabolites were analysed of which seven were detected in the topsoil. Almost all active ingredients were continuously detectable after application until the beginning of next year. Depending on the active ingredient and application date, this could be between 6.5 and 9 months. Atrazine, last applied in 2009 as well as its degradation product atrazine-2-hydroxy, could be detected in soil over the whole monitoring period. Pesticide contents in the field were compared to predicted environmental concentrations (PEC) with min. and max. *DT*_50_ from different sources such as the European Food Safety Agency (EFSA) conclusions or the University of Hertfordshire (2023). The PECs were found to be adequate in comparison to topsoil concentrations from the field. Four active ingredients were detected in the potato tubers, but none of them exceeded the maximum residue level in Switzerland.

## Deficits and needs identified by presenters for a pesticide network for Central and South America

After 16 contributions in a two-day workshop (10/11 May 2023; Table 1), the presenters discussed and identified deficits, needs, interests and opportunities to establish a **pesticide network for Central and South America**. According to them, the lack of awareness of pesticide use (e.g. Fuhrimann et al. 2019b) affects the health and safety of workers applying the chemicals to (mostly) plants. Despite Latin America representing the main agricultural area in the world with a very intense pesticide use, monitoring data of pesticides in soil, surface and groundwaters, food, as well as in humans are missing especially for the notorious herbicide glyphosate. The main reason for this is the difficult analysis of the compound. Moreover, in theses matrices, information about other currently used pesticides such as paraquat, atrazine, imidacloprid, thiamethoxam, linuron, boscalid, chlorpyrifos, and chlorothalonil is scarce and should be therefore monitored as well. Given the little information on mancozeb, it should also be determined in food, e.g. Colombian coffee and waters.

There is also a need to assess retrospectively the risks of pesticides to workers, farmers, people living in vicinities of pesticide intensive crops, consumers and different environmental compartments. That should enable authorities to withdraw or limit in “short time” the access to formulations on the market containing active ingredients posing risks. A general deficit is communication not being state of the art in bottom-up as well as top-down ways, including for instance the information status and the teaching of workers, farmers or the public how to correctly use and apply pesticides, thus avoiding exposure or of the decision makers to restrict or limit HHPs.

Pollinators suffer from multiple stressors not least due to pesticides, especially neonicotinoids and, as a consequence, decline (Douglas et al. 2022; Walker and Wu 2017). Hence, alternative pesticides are badly needed. On the technical side, the different analytical methods to determine residues of active ingredients and transformation products in matrices of concern should be harmonized among laboratories. In summary, consensus existed among the workshop participants that the above deficits could be effectively identified, addressed and amended by means of a joint Latin American pesticide network.

## Aims of a future pesticide network for Central and South America

Based on the above deficits and needs, future actions and goals of the **pesticide network for Central and South America** to alleviate negative impacts of the use of pesticides shall be:

1. Instruct users on the best management practices on how to protect themselves, how to dose and apply pesticides and provide personal protective equipment. Additionally, the personal protective equipment for workers handling pesticides in the tropics should be climatized or vented so that it is bearable to wear.
2. Monitor currently used pesticides of concern, first and foremost glyphosate in different matrices such as the environment, food and consumers. Survey biodiversity.
3. Assess the effects on humans retrospectively by evaluating the risks emanating from the use of pesticides with a multicriterial method as a kind of “post-registration” in order to (continues with 4)
4. Supply authorities and decision makers with the necessary information for active ingredients to be withdrawn from or limited on the market.
5. Avoid synthetic pesticides and advertise and propagate the use of alternative pesticides as for instance extracts from custard apple, chili, eucalyptus, tobacco or castor-oil plant, mixtures with minerals and/or soaps as basis, use of bacteria, fungi, viruses or synthetic pesticides with less risks to humans and the environment (Barrientos 2021; Ramirez-Muñoz 2021, Willis 2016) (authors of these reports are Fernando Bahena-Juárez, Mexico, and Fernando Ramírez-Muñoz, Costa Rica, who presented their work at the SISA; Table 1). In any case, teach pros and cons of (synthetic) pesticides in school and also educate how to apply good agricultural practice to farmers.
6. Harmonize analysis of pesticides in the different Latin American countries, as good as possible in each of the different matrices, such as soil, water, food and humans.
7. Check whether a future **pesticide network for Central and South America** could best achieve its goals by forming within/joining an existing network (Table 2) or when set up independently. Aspects to consider here are visibility, opportunities for outreach, accessibility to stakeholder such as decision makers, authorities, industry, potential synergies such as existing infrastructure, logistics or financial support.

**Table 2:** List of existing networks, their websites and main focuses. The first five (#1–5) networks focus exclusively on pesticides and related issues. While the Pesticide Action Network (PAN, #1) International is global, RAP-AL (#2) and RAP-AM (#3) are PAN organizations of Latin America and Mexico, respectively. From #1 to #3, aims and actions get more and more specific. Although not in the PAN, this also holds for the Brazilian Campaign against pesticides, which has specific actions on its agenda (#4). The SETAC interest group (IG, #5) of pesticides acts on the scientific level and assesses the impacts of plant protection products in the environment for regulatory purposes. #6–9 are organizations focussing on chemical pollution, toxic chemicals or chemical safety where pesticides are part of their agenda. They concentrate on providing information in general (UNEP, #6), or for policy makers more specifically (IPEN, #7 and SAICM, #8), and scientific knowledge about issues of chemical pollution to decision makers and the public (IPCP, #9)

No	Website	Main focus and additional comments
1	Pesticide action network international (PAN) https://pan-international.org	PAN International was founded as a global network in 1982 in response to the fundamentally international nature of the pesticide problem. The network now links over 600 groups, institutions and individuals in more than 90 countries. Its members work through five independent, collaborating regional centres. PAN International is a network of grassroots organizations (PAN Latin America (see #2), PAN Europe, PAN North America, PAN India), and as such, it does not have a central office. In each region, the PAN Regional Centre coordinates a large network of like-minded groups and builds alliances with other networks, and these make up the global PAN. Campaigns, activities and relevant objectives are decided by the Regional Centre in collaboration with its network. This gives each region the ease to be able to respond quickly and coordinate efficiently the priorities set by its member organizations.
2	Pesticide action network of Latin America (Red de Acción en Plaguicidas y sus Alternativas para América Latina, RAP-AL) https://rap-al.org	RAP-AL is the PAN of Latin America and a network of organization, institutions and associations opposing the massive and indifferent use of pesticides. RAP-AL promotes practical alternatives to synthetic pesticides for an ecologically sustainable, socially just and economically viable development of the Latin American agriculture allowing to achieve the food sovereignty of local communities. The following co-authors of this conference report are members of RAP-AL: 1. Fernando Ramírez-Muñoz (Costa Rica) 2. Fernando Bahena-Juárez (México) 3. Nilda Pérez-Consuegra (Cuba, after COVID-19, activities as well as finances decreased)
3	Pesticide action network Mexico (Red de Acción sobre Plaguicidas y Alternativas en México, RAP-AM) https://www.rapam.org/	RAPAM is a Mexican civil non-profit association to: • Progressively eliminate chemical pesticides that affect human health and the environment • Promote necessary changes in public policies to stimulate pest control in an organic and agro-ecological manner, to protect the rights to a sound alimentation that is also free of transgenic crops for pest control, to achieve the food sovereignty and an environment free of pollutants Belongs to RAP-AL.
4	Campanha Permanente Contra os Agrotóxicos e pela Vida (Brazilian Campaign against Pesticides for life) https://contraosagrotoxicos.org/	Network of academic institutions and civil society in Brazil which aims to denounce cases of accidental and criminal contamination, hazards associated to pesticides and agribusiness and agroecology as a healthy and sustainable way to produce food.
5	Society of Environmental Toxicology and Chemistry (SETAC interest groups) https://www.setac.org/group/environmental-monitoring-of-pesticides.html	SETAC Interest Groups provide a vital forum for professionals in the environmental sciences to advance a scientific topic under the SETAC umbrella, with oversight from governance. The groups’ activities range from sponsorship of platform and poster sessions at SETAC annual meetings to proposing and supporting focused topic meetings, symposia and workshops. They are also instrumental in identifying current scientific topics of interest to the society. The scope of the Interest Group (IG) pesticides is environmental monitoring and post-registration studies related to the use of plant protection products (PPP) in crop protection as related to European regulatory frameworks such as the Water Framework Directive (Directive 2000/60/EC). The IG covers areas of the environmental assessment related to PPP in a regulatory context, including wildlife, terrestrial invertebrates (such as honeybees), aquatic communities, but also the quality of soil and water compartments including ground water.
6	United Nations Environment Programme (UNEP) https://www.unep.org/about-us	UNEP is the leading global authority on the environment and its mission is to inspire, inform and enable nations and peoples to improve their quality of life without compromising that of future generations. UNEP is driving transformational change by drilling down on the root causes of the triple planetary crisis of climate change, nature and biodiversity loss and pollution.
7	The International Pollutants Elimination Network (IPEN) https://ipen.org/	IPEN is a global network forging a healthier world where people and the environment are no longer harmed by the production, use and disposal of toxic chemicals. Over 600 public interest NGOs in more than 120 countries, largely low- and central-income nations, comprise IPEN and work to strengthen global and national chemicals and waste policies, contribute to ground-breaking research and build a global movement for a toxic-free future.
8	Strategic Approach to International Chemicals Management (SAICM) https://saicm.org/	Adopted by the First International Conference on Chemicals Management (ICCM1) on 6 February 2006 in Dubai, SAICM’s approach is a policy framework to promote chemical safety around the world. Its overall objective is the achievement of the sound management of chemicals throughout their life cycle so that by the year 2020, chemicals are produced and used in ways that minimize significant adverse impacts on the environment and human health. Objectives are grouped under five themes: 1. Risk reduction 2. Knowledge and information 3. Governance 4. Capacity-building and technical cooperation 5. Illegal international traffic
9	International Panel on Chemical Pollution (IPCP) https://www.ipcp.ch/	The goal is to collect scientific knowledge about issues of chemical pollution and to provide summaries and interpretations of the available knowledge for decision makers and the public. Groups of chemicals are as follows: pesticides and biocides; pharmaceuticals; industrial chemicals such as solvents, flame retardants and plastic softeners; and unwanted by-products such as polychlorinated dibenzodioxins and furans. Lately, there has been some progress in the science-policy interface. For instance • A white paper was published to contribute further to the sound management of chemicals and waste and to prevent pollution (Interational Panel on Chemical Pollution 2023a) • A response was written regarding the operating principles governing the work of the panel emphasizing the need for explicit inclusion of conflict of interest provisions (Interational Panel on Chemical Pollution 2023b), and • A letter in Science was written by IPCP Board Members and colleagues, highlighting obstacles to scientific input into UNEP processes including those on chemicals and waste (Interational Panel on Chemical Pollution 2023c)

## How to achieve the goals

To level off and to complement the information status of the presenters of the different countries, already existing data and results of goals 1 to 5 could be exchanged between Latin American countries and experiences shared. The following paragraph about a cross-sectional study on the exposure to pesticides of smallholder farms of conventional and organic management in Costa Rica in 2016 (Fuhrimann et al. 2019b) shows that programs about pesticide issues in Latin America already were and are being performed and that the training of workers and farmers is not the end of the story.

Fuhrimann et al. (2019a) revealed that farm workers who received training on pesticide use had weekly pesticide exposure scores of 33% less than those who did not receive training. However, the paper of Staudacher et al. (2020), who extended the study area of Costa Rica to Uganda, reported that despite the smallholder farmers from both countries were aware of the negative health effects of pesticides exposure, the majority of them classified as HHP, < 11% in Costa Rica and < 2% in Uganda reported using personal protective equipment. Workers of conventionally managed farms from Costa Rica (14%) and Uganda (19%) reported disposing pesticide residuals into rivers (Staudacher et al. 2020). The authors concluded that the training of farmers about the pesticide use should be target-group oriented focusing on the farmers’ perception of risk. Future studies should not only focus on proper application practices and protection but also on agronomic measures such as integrating pest management, fostering the professionalization of farmers and promoting the management of pesticide-related impacts beyond applicator health (e.g. run-off into community-streams, spray-drift, consumer health). Preventive efforts such as training should include, besides farmers, other actors along the pesticide value chain. Therefore, future research should focus on pesticide sales, pesticide import policies and end-consumer sales to prevent hazardous chemicals to unqualified buyers (Staudacher et al. 2020).

To achieve goal 6, samples could be exchanged to perform interlaboratory comparisons and ring trials to harmonize methods of chemical analysis. Quality control and quality assurance of environmental samples within Latin America could be improved based on the feedback of the workshop participants. A shared wish of the presenters (Fig. 1; Table 1) was to create a platform of interaction to address issues described above representing a starting point and to enhance collaboration.

To achieve goal 7, the authors would need to approach representatives of the various existing networks (Table 2) to evaluate whether pesticide problems can be brought into, discussed and solved within their respective framework. Alternatively, a new **pesticide network for Central and South America** could be built or other assemblies could be formed (goal 7). For instance, a new **pesticide network for Central and South America** could seek collaboration with RAP-AL, the pesticide action network of Latin America (Table 2, #2) or form a partnership with the pesticide Interest Group within the Society of Environmental Toxicology and Chemistry (SETAC IG; Table 2, #5). Joining an existing network can be a win–win situation for both sides, because additional expert knowledge is integrated, and a larger group can benefit from already established connections. For instance, the International Panel on Chemical Pollution (IPCP), which operates at the environmental science-policy interface (Table 2, #9), can aid to convey information to decision makers about HHP, a class of chemicals not yet extensively addressed by them. Furthermore, joint forces can harmonize the education of pupils and students in Latin America about the pros and cons of pesticides, their use and impact on humans and the environment (goal 5).

The availability of funding from international associations/organizations (Table 2, #1, #6, #7, Food and Agricultural Organization (FAO)), private foundations and European Research Programs is needed not only for pesticide monitoring in various one health compartments but also for surveys and for a broad and systematic dissemination of results to decision makers, consumers and farmers. A good example from the past was the project to phase out HHP and seek alternatives in Costa Rica with IRET (regional institute of studies on toxic substances), PAN UK (Table 2, #1), UNA (Costa Rica’s national University), UNEP (Table 2, #6) and SAICM (Table 2, #8) as participating organizations (Willis 2016). A new network could also profit from the Swiss Action Plan on pesticide risk reduction (BLW 2017; Godbersen et al. 2019), because many of the 50 + measures taken therein can also be useful and applicable within the Latin American context.

## Next steps

The authors see this conference report as a basis to get an overview about pesticide monitoring and related topics in Latin America. As a next step, Miguel Ángel Gonzalez-Curbelo (Table 1, professor at the EAN University in Bogotá, Colombia with a PhD in chemistry and chemical engineering) kindly offered to take the lead in compiling a subsequent review on analysis of HHP in Latin America. The workshop participants, who contributed to this session at the SISA, will gather additional information to identify and/or sharpen objectives for the planning of a new **pesticide network for Central and South America**. Furthermore, each consortium member will think of her/his strengths, weaknesses and options on earlier research programs that can help to decide which organization could be joined and where to seek financial sources. Readers of this article are welcomed to contact any of the co-authors in their respective region on any matter addressed and bring in their ideas.
